# Correlation between deletion of the CDKN2 gene and tyrosine kinase inhibitor resistance in adult Philadelphia chromosome-positive acute lymphoblastic leukemia

**DOI:** 10.1186/s13045-016-0270-5

**Published:** 2016-04-18

**Authors:** Na Xu, Yu-ling Li, Xuan Li, Xuan Zhou, Rui Cao, Huan Li, Lin Li, Zi-yuan Lu, Ji-xian Huang, Zhi-ping Fan, Fen Huang, Hong-sheng Zhou, Song Zhang, Zhi Liu, Hong-qian Zhu, Qi-fa Liu, Xiao-li Liu

**Affiliations:** Department of Hematology, Nanfang Hospital, Southern Medical University, Guangzhou, 510515 China; Guangzhou Air Force Headquarters Hospital, No. 475, Huanshi East Road, Yuexiu District, Guangzhou, 510071 China; Department of Hematology, The Second People’s Hospital of Guangdong Province, Guangzhou, 510317 China; Department of Hematology, Hospital of Guizhou Province, Guizhou, 550002 China

**Keywords:** CDKN2, Acute lymphoblastic leukemia, CD20, Philadelphia chromosome, Tyrosine kinase inhibitors, Deletion

## Abstract

**Background:**

Frequency relapses are common in Philadelphia chromosome-positive (Ph-positive) acute lymphoblastic leukemia (ALL) following tyrosine kinase inhibitors (TKIs). CDKN2A/B is believed to contribute to this chemotherapy resistance.

**Methods:**

To further investigate the association between CDKN2 status and TKI resistance, the prevalence of CDKN2 deletions and its correlation with a variety of clinical features was assessed in 135 Ph-positive ALL patients using interphase fluorescence in situ hybridization (I-FISH).

**Results:**

Results showed that no difference occurred between patients with CDKN2 deletion (44/135) and wild-type patients in sex, age, and complete remission (CR) rate following induction chemotherapy combined with tyrosine kinase inhibitors (TKIs). However, CDKN2 deletion carriers demonstrated higher white blood cell (WBC) count, enhanced rates of hepatosplenomegaly (*P* = 0.006), and upregulation of CD20 expression (*P* = 0.001). Moreover, deletions of CDKN2 resulted in lower rates of complete molecular response (undetectable BCR/ABL), increased cumulative incidence of relapse, short overall survival (OS), and disease-free survival (DFS) time (*P* < 0.05) even though these patients received chemotherapy plus TKIs followed by allogenic hematopoietic stem cell transplantation (Allo-HSCT). In the case of 44 patients who presented with CDKN2 deletion, 18 patients were treated with dasatinib treatment, and another 26 patients were treated with imatinib therapy, and our study found that there were no differences associated with OS (*P* = 0.508) and DFS (*P* = 0.555) between the two groups.

**Conclusions:**

CDKN2 deletion is frequently acquired during Ph-positive ALL progression and serves as a poor prognostic marker of long-term outcome in Ph-positive ALL patients with CDKN2 deletion even after the second-generation tyrosine kinase inhibitor treatment.

## Background

Tyrosine kinase inhibitors (TKIs) were currently used as front line chemotherapy agents in Philadelphia chromosome-positive acute lymphoblastic leukemia (Ph-positive ALL) patients. Although favorable clinical outcome and complete remission (CR) has been reported, TKI resistance demonstrates a higher relapse rate and short survival time. Development of resistance is a continuous clinical challenge [1]. Therefore, exploration of TKI resistance mechanism and its associated new prognostic markers becomes a model of therapy for particular subgroups of patients who have showed no significant benefit from TKI therapeutic trials [2].

CDKN2A/B deletions including tumor suppressor genes INK4A, INK4B, and/or ARF commonly occur in all types of lymphoid malignancies and account for approximately 55 % of adult T-ALL and 30 % of BCP-ALL [3]. Our previous research reported the unfavorable prognostic role of CDKN2 gene deletion in long-term leukemia outcomes [4]. Especially, an association study between CDKN2 deletion and clinical outcomes suggested CDKN2 as a poor prognostic marker, and this has been observed in 29 % of BCR-ABL-positive ALL [5]. Previous studies failed to demonstrate this phenomenon as they were limited by a small sample size and were unable to investigate the correlation between CDKN2 deletion and immunophenotypic or molecular characteristics. Our current study enrolled 135 newly diagnosed patients who were Ph-positive ALL patients in multi-cancer centers, and the prognostic value of the deletion of CDKN2 gene was assessed.

## Methods

### Patient information

From January 2008 to December 2014, 135 de novo patients diagnosed with Ph-positive ALL at the Nanfang Hospital of Southern Medical University, Guangzhou Air Force Headquarters Hospital, the Second People’s Hospital of Guangdong province, and the Hospital of Guizhou Province following standard bone marrow morphologic, cytochemical, immunophenotypic criteria and cytogenetics were included in our study. All patients received the systematic treatment. Furthermore, we assessed factors that may affect the prognosis of the patients such as age, peripheral white blood cell count of primary diagnosis, hepatosplenomegaly, cytogenetics, phenotype, and other clinical data.

### Ethics statement

The study protocol was approved by the Ethics Committee of the Nanfang Hospital of Southern Medical University.

### Immunophenotyping by flow cytometry

Immunophenotyping by flow cytometry allowed for the differentiation of 127 B-ALL patients. All of them were analyzed and reported according to the European Group for the Immunological Characterization of Leukemias (EGIL) criteria [6].

### FISH and probes

**Fig. 1:**
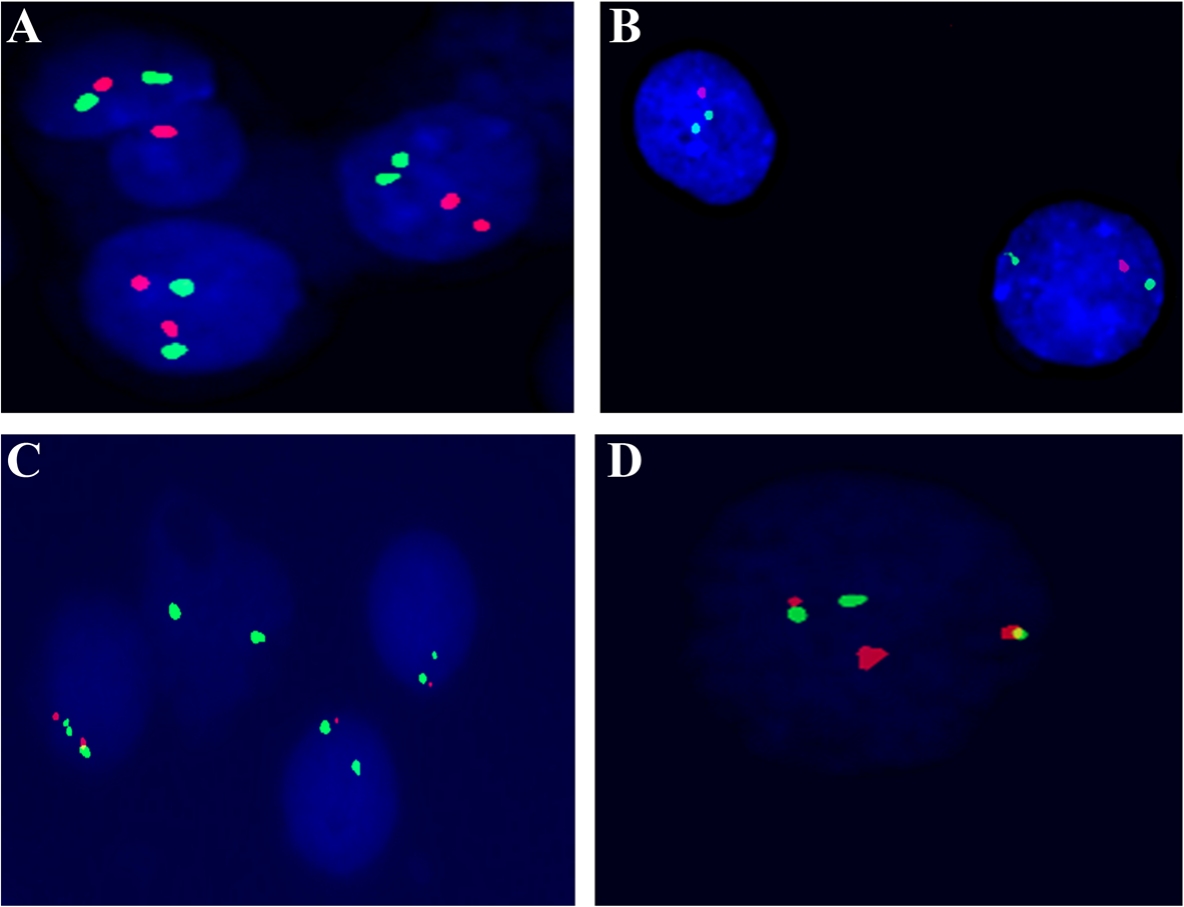
Representative of fluorescence images in situ hybridization. **a** Normal cells presented with double *green* and *red* signals; **b** hemizygous cells presented with loss of one *red* signal; **c** homozygous cells presented with a loss of both *red* signals (p16) and only retained with two *green* signals (chromosome 9); **d** *red* and *green* signal fusion (BCR/ABL+)

We included CDKN2A (encoding p16 and p14) and CDKN2B (encoding p15), the two subunits of CDKN2, in our current study. The deletion of CDKN2 was defined as the loss of CDKN2A or CDKN2B which contained both hemizygous deletion and homozygous deletion genotypes. Interphase fluorescence in situ hybridization (I-FISH) experiments were performed with commercial kits (Cat No. LH009, Cytocell, Cambridge, UK) including two red-labeled CDKN2 probe kits, one red-labeled BCR and one green-labeled and ABL probe kit according to the manufacturers’ protocols (Fig. 1). Bone marrow cells of all patients were collected for detection of CDKN2 (covers a 193-kD region of 9q21.3, extending from 105 kD telomeric of p16 gene to 46 kD centromeric of CDKN2B) and BCR/ABL. We analyzed interphase cells according to the manufacturer’s instructions and the ISCN (2005) criteria [7].

### Real-time quantitative polymerase chain reaction

The BCR-ABL expression levels of BCR-ABL were detected by real-time quantitative polymerase chain reaction (RT-PCR) (Qiagen, Hilden, Germany). ABL was chosen as an internal control. The results were presented with a percentage of BCR-ABL/ABL. It was used to evaluate the complete remission rate of patients following a period of treatment.

### Treatment protocol

According to the National Comprehensive Cancer Network (NCCN) Guideline Version 1.2014 Acute Lymphoblastic Leukemia [8], a 4-week induction therapy (vincristine, daunorubicin or idarubicin, l-asparagines, and prednisone) was given to all patients with a supplementary dose of imatinib 400 mg qd per day or dasatinib 100 mg qd per day once a day. All patients were then treated with consolidation therapy including Hyper CVAD A scheme (cyclophosphamide, vincristine, daunorubicin, and dexamethasone) and alternately Hyper CVAD B scheme (high-dose methotrexate and cytarabine) following complete remission. Our study enrolled 96 cohorts undergoing their first or second remission and who required allogenic hematopoietic stem cell transplantation (Allo-HSCT). The procedure required that all cohorts with diagnosed ALL should undergo central nervous system (CNS) prophylaxis. Follow-up of the ALL cohorts ran till August 1, 2015 (median follow-up: 25.6 months, range: 1.2–78.9 months). Confirmed complete molecular response (CMR) was defined as lower than 0.0032 % [9].

### Statistical analysis

SPSS 17.0 software (SPSS Inc., Chicago, IL, USA) was used to evaluate the statistical difference of categorical variables between patient groups with the Pearson Chi-square analysis and Fisher exact test. Disease-free survival (DFS) was calculated from the date of complete remission to the first relapse. The Kaplan–Meier method and Log rank tests were performed to compare overall survival (OS) between the groups, and a *P* value of less than 0.05 was considered statistically significant.

## Results

### Characteristics of patients

One hundred thirty-five newly diagnosed Ph-positive B-cell ALL patients (age 18–65, median 33.4) were enrolled. The characteristics of the patients are summarized in Table 1. Of the 135 cases analyzed, 44 (32.6 %) patients showed that they were carriers of CDKN2 deletion. No significant differences were observed for age and gender between CDKN2 deletion carriers and non-carriers. The median white blood cell (WBC) count was 54.1 × 10^9^/L (range: 0.8~353.0). However, the initial WBC counts and hepatosplenomegaly rate of CDKN2 deletion were significantly higher than those of patients with no deletion (*P* = 0.012, *P* = 0.006, respectively).

**Table 1:** Patient characteristics (*N* = 135)

Clinical characters	CDKN2 deletion	No CDKN2 deletion	*P* value
Adults (sample size)	44	91	
Male/female	24/20	53/38	0.684
Mean age (years)	33.3 (18–64)	35.4 (18–65)	0.368
WBC count (×10^9^/L)^a^	107.7 (1.6~302.0)	69.8 (0.8~353.0)	0.012
Hepatosplenomegaly^a^	26 (59.1 %)	31 (34.1 %)	0.006
Complete remission	39 (88.6 %)	85 (93.4 %)	0.337
Stem cell transplantation	34 (77.3 %)	62 (68.1 %)	0.272
Relapse^a^	26 (59.1 %)	32 (35.2 %)	0.008

*WBC* white blood cell

^a^Comparison between CDKN2 deletion carriers and non-carriers

### Immunophenotypic analysis

Of the 135 patients analyzed in our study, 127 received immunophenotypic analysis, and the results are summarized in Table 2. CD20 expression was defined as ≥20 % cells that are positive with CD20. Within the subgroup of CDKN2 deletion, 25 of 42 (59.5 %) patients analyzed expressed CD20, and our results showed that there were significant differences between the patients with and without CDKN2 deletion in terms of CD20 expression (*P* = 0.001).

**Table 2:** Immunophenotype comparison between CDKN2 wild-type and deletion

Positive antigens	CDKN2 deletion (*n* = 42)	No CDKN2 deletion (*n* = 85)	*P* value
CD34	40 (95.2 %)	75 (88.2 %)	0.334
HLA-DR	42 (100 %)	81 (95.3 %)	0.301
CD45	20 (47.6 %)	28 (32.9 %)	0.109
TdT	2 (4.8 %)	2 (2.4 %)	0.599
CD117	2 (4.8 %)	1 (1.2 %)	0.254
CD10	40 (95.2 %)	79 (92.9 %)	1.000
CD19	41 (97.6 %)	82 (96.5 %)	1.000
CD20^a^	25 (59.5 %)	24 (28.2 %)	0.001
CD22	22 (52.4 %)	53 (62.4)	0.282
CD13	24 (57.1 %)	61 (71.8 %)	0.099
CD14	0 (0 %)	3 (3.5 %)	0.550
CD15	0 (0 %)	0 (0 %)	N/A
CD33	24 (57.1 %)	34 (40 %)	0.068
CD2	3 (7.1 %)	5 (5.9 %)	1.000
CD3	1 (2.4 %)	0 (0 %)	0.331
CD7	0 (0 %)	4 (4.7 %)	0.301
CD56	4 (9.5 %)	4 (4.7 %)	0.438
CD64	2 (4.8 %)	5 (5.9 %)	1.000
CD11b	3 (7.1 %)	3 (3.5 %)	0.396
cCD79a	38 (90.5 %)	67 (78.8 %)	0.103
cMPO	0 (0 %)	0 (0 %)	N/A

^a^Comparison between CDKN2 deletion carriers and non-carriers

### Effect of CDKN2 deletion on complete molecular response

After the induction treatment, 124 patients achieved CR, and no significant differences in CR rate were observed between patients with or without CDKN2 deletion. Eleven patients who failed to achieve CR died early due to sepsis (*n* = 5), CNS (*n* = 2), or pulmonary failures (*n* = 3). In the subgroup of CDKN2 deletion, 6 of 39 (15.4 %) patients analyzed achieved CMR after the induction treatment, and the CMR rate was lower than the subgroup of the wild-type CDKN2 gene (15.4 versus 32.9 %, *P* = 0.042). After two to three courses of consolidation chemotherapy, 34 patients with CDKN2 deletion and 62 patients with the wild-type CDKN2 gene received allogeneic hematopoietic stem cell transplantation (Allo-HSCT). The CMR rate before Allo-HSCT was higher in the group with the wild-type CDKN2 gene than in the group with CDKN2 deletion (77.4 versus 52.9 %, *P* = 0.013). Three cases out of 34 patients with CDKN2 deletion and four cases out of 62 patients without CDKN2 deletion did not survive during the period of Allo-HSCT. After analyzing the evaluable cases, the differences in CMR rates were not observed after the Allo-HSCT (87 versus 93.1 %, *P* = 0.442) (Table 3).

**Table 3:** Influence of CDKN2 deletion on complete molecular response (CMR)

	Patients (dele/wild)	CDKN2 deletion	Non CDKN2 deletion	*P* value
After induction^a^	39/85	6 (15.4 %)	28 (32.9 %)	0.042
Pre-Allo-HSCT^a^	34/62	18 (52.9 %)	48 (77.4 %)	0.013
Post-Allo-HSCT	31/58	27 (87 %)	54 (93.1 %)	0.442

*Allo-HSCT* allogeneic hematopoietic stem cell transplantation

^a^Comparison between CDKN2 deletion carriers and non-carriers

### The influence of CDKN2 deletion on different TKI treatments

Among 44 CDKN2 deletion patients, 26 cases received imatinib treatment and 18 cases received dasatinib treatment, and our results showed no difference in CR, CMR, and relapse rates between patients who received imatinib and those who received dasatinib treatment. Also, no differences were observed in the OS and DFS (Table 4).

**Table 4:** Influence of CDKN2 deletion by different TKI treatments

	Imatinib (*n* = 26)	Dasatinib (*n* = 18)	*P* value
CR after induction	22/26 (84.6 %)	17/18 (94.4 %)	0.634
CMR after induction	2/23 (8.7 %)	4/16 (25.0 %)	0.205
Transplantation	19/26 (73.1 %)	15/18 (83.3 %)	0.489
Relapse	16/26 (61.5 %)	10/18 (55.6 %)	0.691
OS (median time)	16.5 (1.2–38.7)	18.4 (3–41.9)	0.508
DFS (median time)	12.9 (0–37.7)	14.2 (0–41)	0.555

*CR* complete remission, *Allo-HSCT* allogeneic hematopoietic stem cell transplantation, *DFS* disease-free survival, *OS* overall survival

### Influence of CDKN2 deletion on DFS and OS

**Fig. 2:**
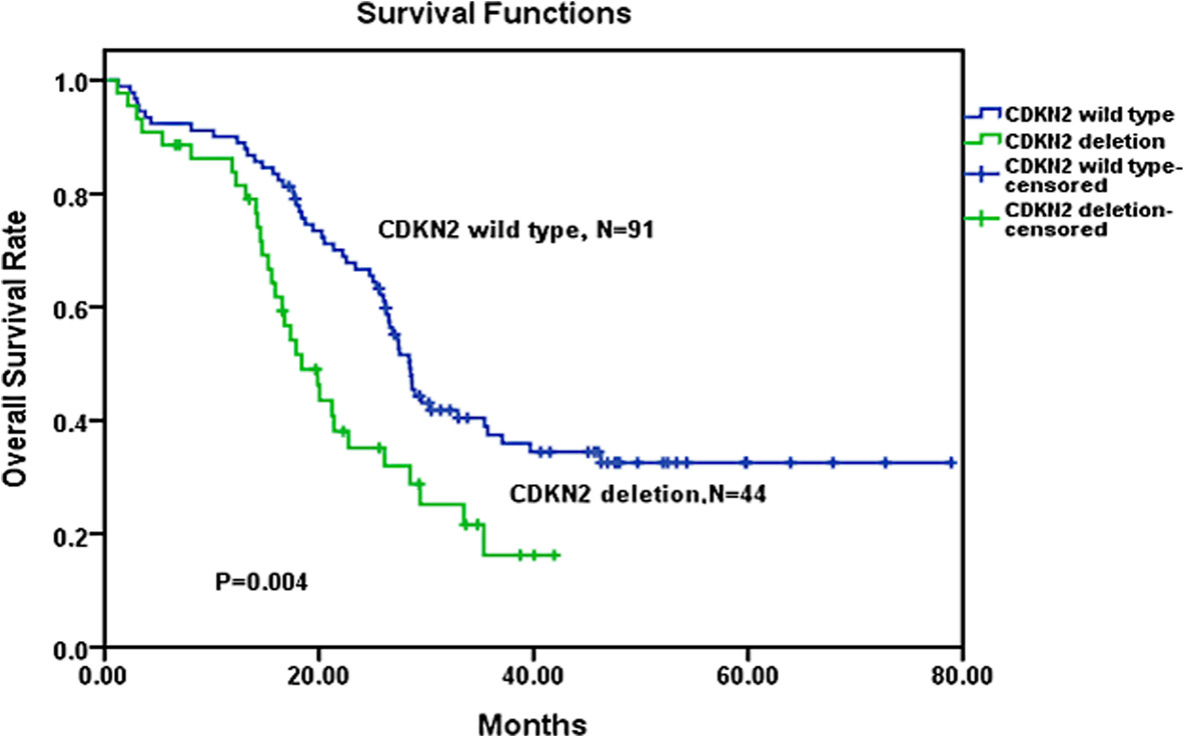
Overall survival comparison among Ph-positive patients. Kaplan–Meier Curve demonstrated significantly a shorter survival time in CDKN2 wild-type patients than in CDKN2 deletion patients (*P* = 0.004)

**Fig. 3:**
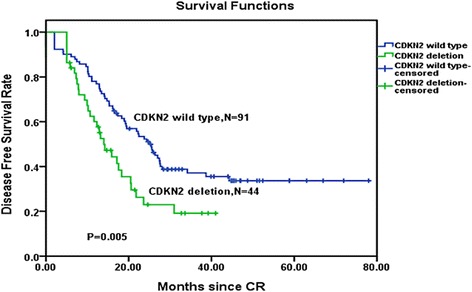
Disease-free survival comparison among Ph-positive patients. Kaplan–Meier Curve demonstrated a significantly shorter disease-free survival time in CDKN2 wild-type patients than in CDKN2 deletion patients (*P* = 0.005). *CR* complete remission

The median follow-up for 135 adults was 25.6 months (1.2–78.9 months). The relapse rate in the CDKN2 deletion subgroup was higher than in the subgroup with no CDKN2 deletion (59.1 versus 35.2 %, *P* = 0.008) (Table 1). OS and DFS curves are shown in Figs. 2 and 3. The estimated 2-year overall survival and 2-year disease-free survival rates by the Kaplan–Meier method for patients with CDKN2 wild-type were 65.5 and 51.1 %, respectively, and for patients with CDKN2 deletion were 35.2 and 23.3 %, respectively. The results revealed that CDKN2 deletion was associated with a significant inferior OS (*P* = 0.004) and DFS (*P* = 0.005).

### Influence of CD20 expression on BCR-ABL-positive B-ALL with CDKN2 deletion

**Fig. 4:**
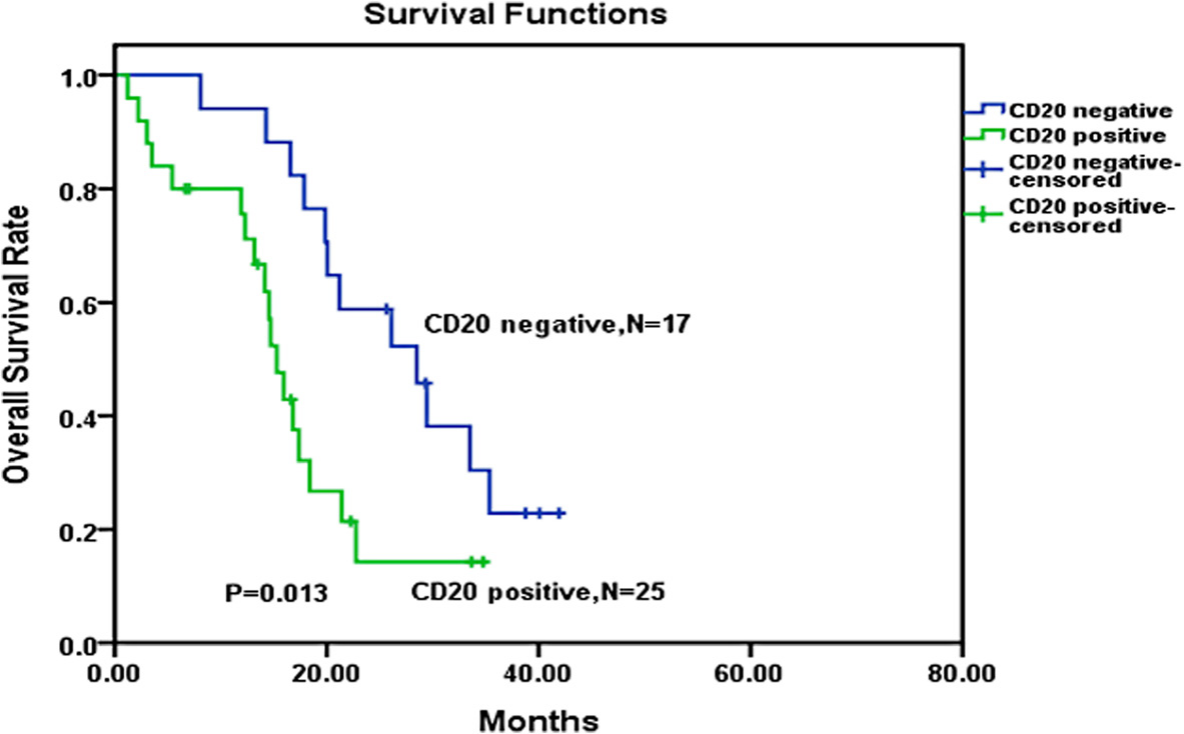
Overall survival comparison among CDKN2 deletion patients received immunophenotypic analysis. Kaplan–Meier Curve demonstrated significantly shorter survival time in CDKN2 deletion patients with CD20-positive patients than in CDKN2 deletion with CD20-negative patients (*P* = 0.013)

**Fig. 5:**
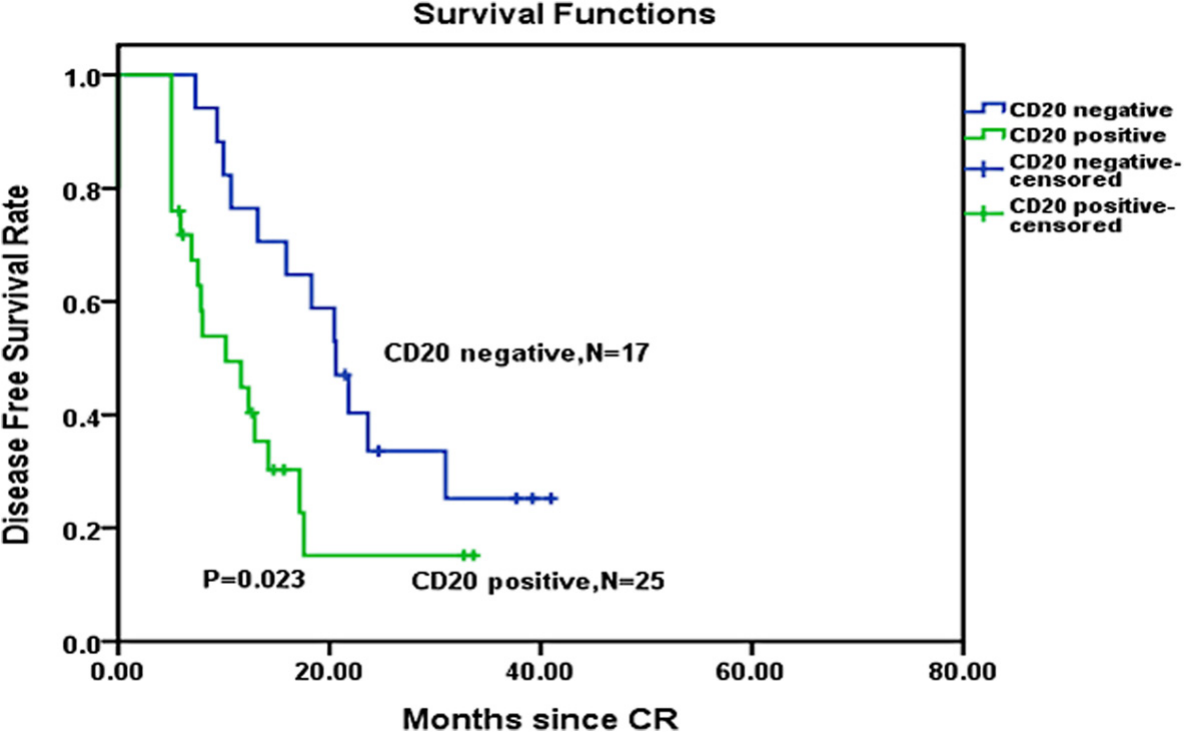
Disease-free survival comparison among CDKN2 deletion patients received immunophenotypic analysis. Kaplan–Meier Curve demonstrated a significantly shorter disease-free survival time in CDKN2 deletion patients with CD20-positive patients than in CDKN2 deletion with CD20-negative patients (*P* = 0.023). *CR* complete remission

Forty-two patients out of 44 CDKN2 deletion patients received immunophenotypic analysis. Analysis of the 42 cases showed that CDKN2 deletion patients with CD20 expression had an inferior OS and DFS than the patients without CD20 expression. OS and DFS curves are shown in Figs. 4 and 5. Our study showed no significant difference in relapse between the CD20-positive and CD20-negative groups (*P* = 0.147).

## Discussion

In the pre-TKI era, the prognosis of BCR-ABL-positive B-ALL has been shown to be extremely unfavorable with 7 years of OS <50 % [10]. Recently, loss of tyrosine kinase receptor in Ph-positive cells was found to result in the development of ALL [11]. The development of TKIs has also been reported to markedly improve the outcome of Ph-positive ALL [12–14]. However, there exists enormous variation in response to chemotherapy with the heterogeneity of biological and clinical outcomes in leukemia patients [15–17]. CDKN2A/B deletion is one of the most common genetic mutations involved in leukemogenesis in Ph-positive ALL cells characterized by recurrent genetic abnormalities along with BCR-ABL fusions [18]. CDKN2A/B genes usually remain undetected in hematopoietic stem cells and begin to activate following blood cell differentiation process in response to potential oncogenic stress [19–21]. The unchanged epigenetic inactivation of CDKN2A/B(INK4-ARF) in differentiated cells will result in an inappropriate self-renewal capacity and leads to malignant transformation. Our current results reported the frequency of CDKN2 deletion as 32.6 % (44/135) in adult Ph-positive B-ALL patients and agreed with the previous observed incidence of CDKN2 deletion in adult patients with the BCR-ABL fusion gene (29 %) [5]. Our results showed that no difference occurred between CDKN2 deletion patients (44/135) and wild-type patients with regard to sex, age, and induction complete remission rate. CDKN2 deletion carriers demonstrated greater white blood cell count, enhanced rates of hepatosplenomegaly, an upregulation of CD20 expression, and a higher relapse rate. It is well known that accelerated tumor cell proliferation could occur as a result of CDKN2A/B deletion due to the direct removal of tumor suppressors and activation of tumor growth factors such as MDM2 and CDK4/6. This may explain why CDKN2 deletion patients present with higher WBC counts, and hepatosplenomegaly is an indicator of a higher tumor load. Currently, research regarding the effect of CD20 in Ph-positive ALL patients has been controversial. Some reports suggested that CD20 expression is not an adverse factor [22, 23]. But, many studies found that expression of CD20 is associated with an increased incidence of a relapse [24], and CD20 upregulation is frequent in patients who suffered later from a relapse [25]. Thus, CD20 expression had an adverse effect on the prognosis in patients diagnosed with B-cell acute lymphoblastic leukemia [26, 27]. For B-lymphoblastic leukemia patients who are Ph-negative, the expression of CD20-positive patients has shown some significant benefit from rituximab therapy, especially towards younger aged patients [28, 29]. Ph-positive ALL cohorts have been prescribed with a similar type of therapy, and these patients most commonly undergo monoclonal antibody therapy. Nevertheless, the adverse expression of CD20 in this particular subtype of ALL has yet to be determined and requires further examination [30]. In our study, the adult Ph-positive ALL patients with CDKN2 deletion had a higher rate of CD20 expression (at a level of at least 20 %), and these patients with CD20 expression had an inferior OS and DFS than the patients without CD20 expression. Hence, CD20 expression may be a cause of the inferior OS and DFS. Together, CDKN2 deletion patients who exhibit a higher CD20 expression could possibly benefit from rituximab treatment. The efficacy of rituximab combination with chemotherapy and its association with the improved survival of patients with ALL needs further research.

Results from the current study revealed that a shorter survival time and a higher recurrence rate was observed in Ph-positive ALL patients with CDKN2 deletion. Previous studies reported similar poor survival as a significant clinical outcome and observed a short relapsed period within 1 year in all of the adult BCP-ALL patients concurrent with the BCR-ABL fusion gene and CDKN2 deletion [5, 31]. In previous studies, CDKN2 deletion was not found in CML-CP patients, but had been detected in part of CML-BC- and Ph-positive ALL patients [32, 33]. Commonly, the CDKN2 gene cluster was silenced in CML-CP progenitors. However, during the process of differentiation, the CDKN2 gene undergoes a series of epigenetic changes in response to BCR-ABL-induced oncogenic signals and consequently stimulates p53 which degrades initial tumor cells via apoptosis. On the other hand, the abnormal progenitors which sustain deletions of the CDKN2 gene acquire an intrinsic self-renewing ability and eventually contribute to the transition to CML lymphoid blast crisis [34]. Previous studies in mouse models, which injected mice with Arf−/− or Arf+/− p210 (BCR-ABL)-positive pre-B cells, demonstrated an aggressive and a higher dose imatinib resistance model [35, 36]. Williams and his team suggested that the BCR-ABL fusion gene and CDKN2 deletion interferes with the tumor suppressor network of Rb and p53, thereby accelerating the self-renewal of leukemia initial cells and enhancing its resistance to the drug [37]. p16(INK4a) or p14(ARF) which are transfected into primary blast cells from CML in blast crisis (CML-BC) and Ph-positive ALL could result in the inhibition of cell proliferation and an increase in cell apoptosis and could further promote sensitivity to imatinib [38]. Recent studies in Ph-positive ALL mouse models showed the attenuation effect of silenced CDKN2 to targeted BCR-ABL kinase inhibitors. Also, CDKN2 inactivation contributes to the prolonged survival of leukemia-initiating cells within the hematopoietic stem cell (HSC) environment and gives rise to the formation of malignant clone cells containing drug-resistant BCR-ABL kinase mutations [35, 36]. Typically, BCR-ABL mutations represent drug resistance in Ph-positive ALL patients, but the deletion of CDKN2 in Ph-positive ALL patients further exacerbates the disease condition and eliminates the favorable outcome of targeted therapy. Together, these mechanisms may explain why additional imatinib or dasatinib therapy presents a poor prognosis in Ph-positive ALL patients with CDKN2 deletion.

## Conclusions

In conclusion, our study assessed Ph-positive ALL patients who presented with CDKN2 deletion and showed that they have a higher rate of CD20 expression. CDKN2 deletion and CD20 expression act as unfavorable prognostic markers for Ph-positive ALL patients despite undergoing tyrosine kinase inhibitor-based therapies. These results provide a better understanding regarding the importance of genetic events, and the study emphasizes a need to pay attention to the Ph-positive B-ALL patients with CDKN2 deletion who could potentially benefit from anti-CD20-directed immunotherapy.

### Consent for publication

All participants in the study signed a written consent form to permit publication of the individual data.

## Abbreviations

Allo-HSCT: allogenic hematopoietic stem cell transplantation
ALL: acute lymphoblastic leukemia
CML-BC: CML in blast crisis
CMR: complete molecular response
DFS: disease-free survival
HSC: hematopoietic stem cell
I-FISH: interphase fluorescence in situ hybridization
OS: overall survival
Ph-positive: Philadelphia chromosome-positive
RT-PCR: real-time quantitative polymerase chain reaction
TKIs: tyrosine kinase inhibitors
